# Design, Implementation, and Evaluation of Anesthesia Students’ Clinical Competency Based on the Virtual Objective Structured Clinical Examination

**DOI:** 10.5812/aapm-155251

**Published:** 2025-01-18

**Authors:** Arghavan Afra, Shima Seneysel Bachari, Maryam Ban

**Affiliations:** 1 Department of Nursing, School of Nursing, Abadan University of Medical Sciences, Abadan, Iran; 2 Department of Anesthesia, School of Nursing, Abadan University of Medical Sciences, Abadan, Iran

**Keywords:** Evaluation, Clinical Competency, Objective Structured Clinical Examination, Virtual Objective Structured Clinical Examination

## Abstract

**Background:**

The objective structured clinical examination (OSCE) is an appropriate method for assessing clinical competency among students in universities worldwide. With nowadays technological advances, there is a growing interest in virtual OSCEs (VOSCEs).

**Objectives:**

The present study aimed to design, implement, and evaluate a VOSCE for assessing anesthesia students’ clinical competency.

**Methods:**

This quasi-experiment study was conducted in six phases: (1) Defining the exam’s specifications and design, (2) determining validity and reliability, (3) setting up and conducting a pilot VOSCE, (4) familiarizing students with the VOSCE, (5) administering the exam, and (6) evaluating and providing feedback. Seventy-five senior anesthesia students from Abadan University of Medical Sciences were selected by census and participated in this study between 2021 and 2023. The scores of virtual and in-person OSCEs were compared, and the correlation between the two exams was investigated. At the end of each semester, students participated in a survey related to the VOSCE. The exam results and survey data were presented at the faculty’s educational development office meetings, and suggestions for amending and eliminating shortcomings were considered in the following semester. Data were analyzed using SPSS 20 by calculating means, standard deviations, and Pearson’s correlation.

**Results:**

The students’ mean scores in the virtual and in-person OSCEs were 17.68 and 16.75, respectively. No significant difference was observed between the scores of the two exams. The total score of the VOSCE had a direct and significant correlation with the in-person OSCE (r = 0.861, P < 0.001), and this correlation was also observed in all stations of both exams (P < 0.05). Student surveys indicated that the VOSCE fostered a sense of empowerment, self-confidence, and enhanced learning, causing students to express strong agreement with its continuation in the future.

**Conclusions:**

The VOSCE can be an appropriate substitute for or an integral part of the in-person OSCE. It is recommended that educational planners and instructors develop this exam as a new assessment method. Given advances in technology and the requirement for improving the quality of virtual exams, professors need to be empowered in the field of modern electronic assessment methods.

## 1. Background

The objective structured clinical examination (OSCE) is an integral part of evaluation methods in medical sciences (1). In recent years, there has been a growing interest in virtual OSCEs (VOSCEs). Initially, the VOSCE was introduced as an innovative solution that allowed remote learners to participate in these assessments (2, 3). Since their introduction in the early 2000s, VOSCEs have been used under various names, such as electronic OSCE (e-OSCE), tele-OSCE, and web-OSCE, and specifically developed to assess the skills required for telecare (4). Although these exams have been fully welcomed by both professors and students and have shown comparable results to conventional OSCEs, they have not become a common assessment method since their advent (3), partly because of students’ uncertainties about the ability of these methods to fully convey their qualities and examiners’ difficulties in assessing clinical skills virtually (5).

However, e-OSCEs can provide a basis to replace traditional examinations with virtual assessment systems and attractive electronic devices (6). The use of e-OSCE reduces administration costs, accelerates the provision of feedback, and enables students to learn more quickly and easily (7). With e-OSCE, examiners can easily use their electronic devices, such as mobile phones and tablets, and document their comments directly (8). Further, e-OSCEs reduce post-examination manual compilation of data, minimize potential score management errors, and enable provision of quick and efficient feedbacks. Some challenges in this area include extensive pre-examination data feeding and hardships in handling the technology by examiners (9). Results of studies indicated that VOSCEs were effective for both learning and assessing clinical competency in students (10). The use of virtual assessment methods not only makes students aware of their strengths and weaknesses in knowledge and clinical skills but also motivates them to enhance their strengths and eliminate their weaknesses. By using virtual clinical skill assessment exams, students’ self-awareness of their educational needs can be enhanced before entering the work field. Another objective of administering virtual clinical competency exams is to assess students’ capabilities in the skill, educational, and communication domains in order to effectively fulfill their roles and responsibilities before entering clinical practice (11, 12). Despite the aforementioned, studies comparing VOSCEs with traditional OSCEs are limited (4).

Anesthesia is a field heavily reliant on practical and clinical skills. The first mistake in surgery can be the last, leaving behind an irreparable injury (13). Therefore, it is necessary to evaluate students’ competency and abilities before they enter the workplace (14). Each country has its own standardized student assessment methods, learning objectives, and testing facilities, according to which the implementation of e-OSCEs can be facilitated or restrained. This electronic test can develop clinical skills and is easily accessible to students who intend to engage with clinical practice (15).

## 2. Objectives

The need for an e-OSCE that can realistically demonstrate the skills of anesthesia students was the initial idea for this study. This study examined the usefulness and efficiency of e-OSCE in assessing the clinical competence of anesthesia students and compared the performance of anesthesia students participating in traditional and electronic OSCEs. Our data can be used as a valuable resource for other clinical disciplines. Given the integration of technology into higher-education teaching and assessment, as well as the significance of assessing the capabilities of senior anesthesia students for hospital work, the current study was conducted to design, implement, and evaluate a virtual clinical competency exam for anesthesia students.

## 3. Methods

The present research was a quasi-experiment study conducted on 75 senior anesthesia students between 2021 and 2023. Participants were recruited using the census sampling method. The design, implementation, and evaluation of this research were carried out in six phases.

### 3.1. Phase 1: Defining the Exam’s Specifications and Design

A selected team consisting of the members of the exam evaluation committee and the clinical skills committee, along with the faculty dean and vice-chancellor of education, as well as the faculty members of the anesthesia department, participated in the design of the OSCE. After brainstorming sessions, the number of stations, exam topics, score distribution, evaluation methods, and checklists were determined. Subsequently, the exam specification table and scoring method for each station were formulated, and a scenario for each station was designed. The scenarios for each station were initially determined by the relevant instructor and then reviewed by the selected team. Checklists and scenarios were extracted and designed based on the learning objectives of the course and the primary sources aligned with the curriculum. Considering the learning requirements of students and available resources, seven stations were identified for the exam, including communication, professional ethics, reporting, equipment and devices required for each type of surgeries, familiarity with job duties, cardiopulmonary resuscitation, and clinical examination. To allow for review and comparison, a traditional in-person OSCE was carried out in parallel to the VOSCE with the same number of stations and similar scenarios.

### 3.2. Phase 2: Determining the Exam’s Validity and Reliability

To assess content validity, the checklists and scenarios designed in the previous phase were reviewed and finalized by 10 faculty members. The evaluation committee analyzed and reviewed the questions (structure, taxonomy, and coverage of educational objectives, difficulty coefficient, and clarity). In order to determine reliability, Cronbach's alpha coefficient, correlation between stations’ scores, and correlation between the total score and each station’s score were calculated. Subsequently, the scenarios were revised and refined.

### 3.3. Phase 3: Setting up and Conducting a Pilot Virtual Objective Structured Clinical Examination

The designed and agreed-upon scenarios with various types of questions, including multiple-choice questions, patient management problem (PMP), key feature (KF), clinical reasoning problem (CRP), and virtual puzzle exams, were uploaded and configured in the Faradid electronic exam system. The total duration of both the in-person and virtual exams was 70 minutes, with 3 - 5 minutes allocated to each station. During the in-person exam, after the allotted time, the student was directed to the next station, and in the virtual exam, the ability to answer questions was deactivated, and the next question was displayed. The exam was conducted in several rounds as a pilot with the participation of several faculty members to identify and resolve problems. A member of the selected team was designated as the coordinator for the exam day.

### 3.4. Phase 4: Familiarizing Students with the Objective Structured Clinical Examination

In this phase, two webinars were held to explain how the exam would be administered and provide a guide for participating in the exam. The students’ questions and ambiguities were also answered. Then, the exam date was announced to the students via the website and information channels in social media.

### 3.5. Phase 5: Administering the Exam

Students were randomly assigned to either the in-person or virtual exam. Both exams consisted of 7 similar stations with different but equivalent scenarios. The in-person exam was implemented at the clinical skills center, and the virtual exam was held using the Faradid electronic exam system. Senior anesthesia students who had completed all their theoretical and internship courses were eligible to take the exam. The exclusion criterion was failure to pass any of the aforementioned courses or lack of attending the orientation webinar. Students were authenticated using personal information, such as their national ID numbers and student IDs. In the case of any technical issues during the exam, students could contact the university’s information technology (IT) department, and the responsible official would resolve the problem as soon as possible. After the exam time ended, each station’s answer sheet was sent to the professor who designed the questions for evaluation, and the exam results were announced within 24 hours. The passing score for each station was achieved by correctly answering 50% of the questions, set by the faculty members participating in study design. For example, in the basic clinical skills station, the necessary equipment and steps for sterile dressing were provided to students in a disorganized manner, and they were asked to organize the steps in the form of a puzzle. In the cardiopulmonary resuscitation station, a scenario of a patient with cardiopulmonary arrest along with an electrocardiogram was provided to students, and they were asked to identify the necessary actions. In the anesthesia monitoring station, a one-minute video showing the recorded information of a patient with a spinal anesthesia emergency was displayed for the student on a monitor, and they were asked to specify the necessary anesthesia procedures.

### 3.6. Phase 6: Evaluating and Providing Feedback

**Figure 1.**
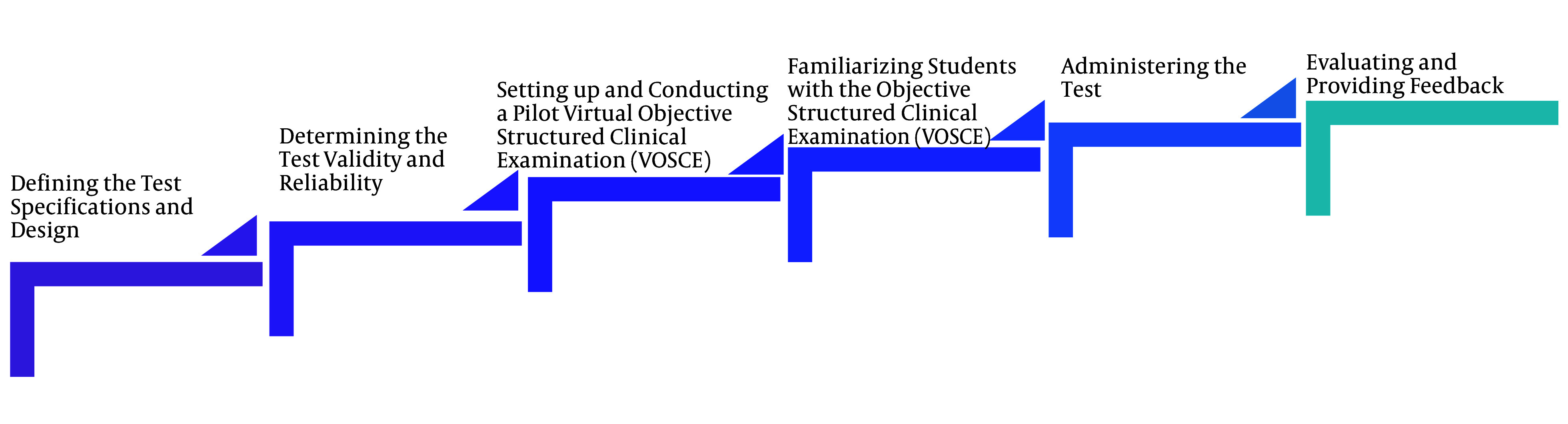
Summary of the design, implementation, and evaluation phases of the virtual objective structured clinical examination (VOSCE)

The final scores of the students participating in the in-person and virtual clinical competency exams were compared. The results of this evaluation were presented at the meetings of the faculty’s Education Development Office (EDO) after each time of implementation to examine its strengths and weaknesses. Colleagues’ suggestions for improvement and rectification of deficiencies were considered for the following semester. Additionally, at the end of each semester, students’ perspectives on the impact of the VOSCE on their learning and satisfaction were assessed using an online questionnaire. Figure 1 shows the steps of designing, implementing, and evaluating the VOSCE.

After data collection, the data were entered into SPSS20. Descriptive analysis was carried out through calculating frequency percentages, means, and standard deviations (SD). Pearson correlation coefficient was employed to examine the relationships between the scores of different stations in the OSCE and VOSCE at the significance level of P < 0.05.

## 4. Results

Seventy-five anesthesia students participated in this exam. Of these, 52 (69.33%) were female, and 23 (30.67%) were male. The mean scores for the virtual and in-person exams were 17.68 and 16.75, respectively, showing no statistically significant difference (Table 1). 

**Table 1.** Comparison of the Results of Virtual and In-person Clinical Competency Exams for Students

Groups	Minimum-Maximum Score	Number of Failing	Mean ± SD Score	P-Value
**Virtual exam**	12.4 - 18.9	0	17.68 ± 2.18	> 0.05
**In-person exam**	11.8 - 18.3	1	16.75 ± 3.46	

In order to examine the correlation between stations’ scores in the two tests, as well as the total score and each station score, Pearson correlation was used at a confidence level of 99% and a significance level of 0.05. The findings showed that the total scores of the VOSCE and in-person OSCE had a direct and significant correlation with each other (r = 0.861, P < 0.001). This relationship also existed between all stations’ scores of the VOSCE and in-person OSCE (P < 0.05), including communication (r = 0.737, P < 0.002), professional ethics (r = 0.784, P = 0.001), reporting (r = 0.831, P < 0.001), equipment and devices (r = 0.770, P < 0.001), description of duties (r = 0.735, P < 0.002), cardiopulmonary resuscitation (r = 0.855, P < 0.001), and clinical examination (r = 0.748, P = 0.001) stations. Appendix in the Supplementary File shows the correlation between these stations in the VOSCE and in-person OSCE.

At the end of each semester, students' views on the way the test was administered, the effectiveness of the test, and their views on taking the test in future were surveyed. The findings showed that 80% of the students believed that the questions asked aligned with the syllabus of the courses taught; 92% of them believed that the VOSCE contributed to their senses of empowerment and self-confidence; 90% stated that administering the virtual exam enhanced their acquisition of practical skills in their field of study; 91% believed that many fields in the virtual exam could replace the in-person exam in subsequent years, and 89% of the students strongly agreed with the continuation of the exam in the future. Table 2 presents the results of the survey regarding the VOSCE.

**Table 2.** Students’ Perspectives on Different Dimensions of the Virtual Objective Structured Clinical Examination ^a^

Dimensions and Items	Very Much	Much	Moderate	Low	Very Low	No Answer
**Exam administration procedures**						
Before administering the exam, I was fully aware of its administration procedure.	37	41	6	6	10	0
The exam’s duration was appropriate.	54	22	7	6	11	0
The questions asked aligned with the syllabus of the courses taught.	50	30	16	3	1	0
The questions asked were consistent with the in-person exam.	64	20	9	3	3	1
**Effects of administering the exam**						
Administering this exam increases students’ motivation.	80	16	0	1	0	3
This exam has caused students to appropriately review forgotten course materials.	81	14	0	4	0	0
This exam has enabled students to assess their readiness to enter the hospital.	80	13	3	3	1	0
This exam has improved students’ performance in their clinical skills at the hospital ward.	76	7	7	4	3	0
Administering the exam has improved students’ acquisition of skills in their field of study.	76	14	4	4	1	0
This exam has made me feel more empowered and confident.	73	19	3	4	1	0
**Dimension and Items**	**Completely Agree**	**Agree**	**No Idea**	**Disagree**	**Completely Disagree**	**No Answer**
**Administering the exam in the coming years**						
The continued administration of this exam in future years will be beneficial to students.	75	14	4	7	0	0
Feedback from this exam should be effectively communicated to professors to encourage its implementation in future years.	63	19	7	8	3	0
Many fields in the virtual exam could potentially replace the in-person exam in future years.	79	12	7	1	1	0
Students should be surveyed about exam administration prior to the process.	58	27	4	4	3	4

^a^ Values are expressed as (%).

Experts’ comments and suggestions on the strengths and weaknesses of the exam were discussed in the faculty’s EDO meetings. Several notable points could be mentioned regarding the strengths of the VOSCE. For example, the planning, preparation, and administration of an in-person OSCE require significant time, cost, and human resources, which were minimized in the VOSCE. Additionally, most students would experience high levels of stress during in-person OSCEs; however, according to this survey, stress levels decreased in the virtual exam, resulting in better performance.

## 5. Discussion

In this research, the clinical competency exam for the anesthesiology program was conducted virtually using the Faradid electronic exam system between 2021 and 2023. The exam’s questions emphasized the clinical and practical aspects of each station. The results of the virtual exam showed no significant differences compared to the mean scores obtained in the in-person exam. Furthermore, a substantial percentage of the students expressed support for the continuation of virtual exams in the future, favoring the possibility of replacing in-person exams with virtual formats.

Bouzid et al., in a study at the Medical School of the Université de Paris Citeg, compared remote versus live evaluations and assessed the factors associated with score variability during e-OSCEs. The findings showed that remote evaluation was as reliable as live evaluations for e-OSCEs (16). In Arrogante et al.’s research, the clinical competency of nursing students during the COVID-19 pandemic was assessed using a standardized VOSCE in Paris, demonstrating that the VOSCE was a cost-effective method and as successful as the in-person exam (17). In Iran, Tolabi and Yarahmadi conducted a virtual clinical competency exam on 42 senior nursing students at Khorramabad University of Medical Sciences in 2021 and reported that this exam was comparable to the in-person clinical competency exams previously held at the same faculty in terms of results, indicating that the virtual exam could replace the in-person format (14). The results of these studies aligned with those of the current study, pointing to the effectiveness of virtual assessment methods. Hence, VOSCEs can be effectively employed when in-person exams are not possible, offering an appropriate or complement substitute for traditional exams.

Concurrently with the COVID-19 pandemic, Hopwood et al. conducted a VOSCE at UCL Medical School in London to assess the competencies of senior medical students. All stakeholders provided positive feedback based on their experiences, with 94% recommending the virtual exam (18). Gamble et al. enrolled medical students to determine whether OSCE training could replace or be used as a complement strategy for in-person training in the post-COVID educational era and demonstrated improvements in all areas of education, including self-confidence, communication, history taking, diagnosis, and clinical reasoning. The results of the recent study recommended that VOSCE training should be employed to complement in-person training (19). In Alshammari’s study, the implementation of e- OSCE was evaluated during the COVID-19 lockdown in a pharmacy faculty in Saudi Arabia, to assess students’ experiences and satisfaction with the implementation of e- OSCE as a potential replacement for traditional OSCE. All the study participants (100%) agreed that e-OSCE saved time; 62.5% noted that e-OSCE was easy to use and grade. Overall, the study showed that e-OSCE was effective in facilitating the assessment process and could be used effectively during certain and uncertain times (20). The results of these studies align with our observations, pointing to the high satisfaction of students with virtual assessment methods.

### 5.1. Conclusions

Clinical competency assessment for anesthesiology students was conducted using the VOSCE method for the first time at Abadan University of Medical Sciences. Based on the results of this study, VOSCE could be an appropriate substitute for in-person exams or at least be implemented as a part of it. Students also expressed high satisfaction with the idea of administering virtual exams in subsequent years. The current research also evaluated students’ capabilities in various domains of knowledge, critical thinking, and clinical judgment to effectively and successfully fulfill their professional duties before entering the clinical setting. It is recommended that workshops and training courses be held for professors to improve the quality of virtual exams, and that medical universities provide virtual learning centers with up-to-date and high-quality infrastructure, materials, and resources. Comprehensive national studies, including qualitative and quantitative research, are needed regarding the implementation and evaluation of VOSCEs in various academic fields. Our VOSCE reflected a new approach in medical education that could open the door to implementing a new approach to assessing medical students' practical skills. However, the enigma of assessing physical and procedural skills remains a challenge, and further technology-based innovations using artificial intelligence and augmented reality are awaited.

### 5.2. Strengths

The strengths of the virtual exam implemented include familiarizing students with modern assessment methods and virtual examination systems. Furthermore, integrating technology into clinical competency assessment allows for the design of a wider variety of questions.

### 5.3. Limitations

The weaknesses of the virtual exam included its administration in only one field of study and the small number of students participating in the experiment.

aapm-15-1-155251-s001.pdf

## Data Availability

The dataset presented in the study is available on request from the corresponding author during submission or after publication.
