# Supplementary material for: Design, Implementation, and Evaluation of Anesthesia Students’ Clinical Competency Based on the Virtual Objective Structured Clinical Examination
Source: Anesth Pain Med. 2025 Jan 18;15(1):e155251. doi: 10.5812/aapm-155251 (PMC12125663; doi:10.5812/aapm-155251)
Supplement: aapm-15-1-155251-s001.pdf [file aapm-15-1-155251-s001.pdf]

Appendix. Pearson's Correlation Coefficient (r) Between Scores Obtained at the Stations of the Virtual and In-person Exams

**Variables**	**VOSCE**	**Communication**	**Professional Ethics**	**Reporting**	**Equipment and devices**	**Description of duties**	**Cardiopulmonary Resuscitation**	**Clinical Examinations**	**In-person OSCE**	**Communication**	**Professional Ethics**	**Reporting**	**Equipment and Devices**	**Description of Duties**	**Cardiopulmonary Resuscitation**	**Clinical Examinations**
**VOSCE**																
r	-															
P	-															
**Communication**																
r	0.911	-														
P	= 0.001	-														
**Professional ethics**																
r	0.903	0.846	-													
P	< 0.01	< 0.002	-													
**Reporting**																
r	0.868	0.732	0.806	-												
P	< 0.01	= 0.001	< 0.001	-												
**Equipment and devices**																
r	0.902	0.816	0.792	0.803	-											
P	= 0.001	< 0.001	< 0.004	< 0.001	-											
**Description of duties**																

r	0.808	0.717	0.701	0.687	0.695	-										
P	< 0.001	= 0.004	< 0.002	< 0.004	< 0.004	-										
**Cardiopulmonary resuscitation**																
r	0.836	0.689	0.665	0.707	0.762	0.746	-									
P	< 0.001	< 0.004	< 0.004	< 0.002	= 0.001	< 0.002	-									
**Clinical examinations**																
r	0.899	0.783	0.812	0.735	0.799	0.751	0.811	-								
P	= 0.001	< 0.004	< 0.001	< 0.002	< 0.002	= 0.004	< 0.001	-								
**In-person OSCE**																
r	0.861	0.851	0.822	0.711	0.748	0.748	0.699	0.839	-							
P	< 0.001	< 0.002	< 0.001	< 0.002	= 0.001	< 0.002	< 0.01	< 0.001	-							
**Communication**																
r	0.746	0.737	0.681	0.622	0.699	0.660	0.639	0.740	0.870	-						
P	< 0.002	< 0.002	= 0.004	< 0.001	< 0.01	< 0.002	< 0.004	< 0.002	< 0.001	-						
**Professional ethics**																
r	0.851	0.784	0784	0.721	0.749	0.793	0.722	0.804	0.910	0.785	-					
P	< 0.002	< 0.002	= 0.001	< 0.001	< 0.002	< 0.002	< 0.001	= 0.004	< 0.01	< 0.002	-					

**Reporting**																
r	0.789	0.802	0.763	0.831	0.680	0.701	0.683	0.768	0.868	0.740	0.805	-				
P	< 0.002	< 0.01	< 0.001	< 0.001	< 0.01	< 0.001	< 0.004	< 0.002	< 0.001	< 0.002	< 0.001	-				
**Equipment and devices**																
r	0.829	0.789	0.779	0.747	0.770	0.717	0.643	0.825	0.897	0.817	0.845	0.792	-			
P	< 0.001	< 0.001	< 0.002	< 0.002	< 0.001	= 0.001	< 0.001	< 0.001	< 0.001	< 0.001	< 0.001	< 0.002	-			
**Description of duties**																
r	0.676	0.692	0.669	0.578	0.560	0.735	0.506	0.626	0.778	0.566	0.622	0.594	0.619	-		
P	< 0.001	< 0.002	< 0.002	= 0.004	< 0.004	< 0.002	< 0.004	< 0.004	< 0.002	< 0.004	= 0.001	< 0.001	< 0.004	-		
**Cardiopulmonary resuscitation**																
r	0.741	0.719	0.686	0.700	0.615	0.700	0.855	0.769	0.699	0.658	0.697	0.699	0.679	0.784	-	
P	< 0.002	= 0.001	< 0.001	< 0.002	< 0.01	< 0.002	< 0.001	< 0.002	< 0.002	< 0.002	= 0.004	< 0.004	< 0.004	< 0.002	-	
**Clinical examinations**																
r	0.808	0.812	0.821	0.812	0.812	0.736	0.786	0.748	0.891	0.918	0.899	0.906	0.896	0.873	0.793	-
P	< 0.001	< 0.001	< 0.002	< 0.001	< 0.001	= 0.004	< 0.002	= 0.001	< 0.001	< 0.001	< 0.01	< 0.001	< 0.002	< 0.001	< 0.002	-
